# The role of osmolality in saline fluid nebulization after tracheostomy: time for changing?

**DOI:** 10.1186/s12890-016-0342-x

**Published:** 2016-12-09

**Authors:** Zunjia Wen, Chao Wu, Feifei Cui, Haiying Zhang, Binbin Mei, Meifen Shen

**Affiliations:** 1 Nursing School of Soochow University, Su Zhou, People’s Republic of China; 2 Neurosurgery Department of First Hospital Affiliated to Soochow University, No. 188 Shizi Street, Gusu District, Su Zhou, Jiangsu Province People’s Republic of China

**Keywords:** Care, Complication, Nebulization, Osmolality, Saline, Tracheostomy

## Abstract

**Background:**

Saline fluid nebulization is highly recommend to combat the complications following tracheostomy, yet the understandings on the role of osmolality in saline solution for nebulization remain unclear.

**Objectives:**

To investigate the biological changes in the early stage after tracheostomy, to verify the efficacy of saline fluid nebulization and explore the potential role of osmolality of saline nebulization after tracheostomy.

**Methods:**

Sprague-Dawley rats undergone tracheostomy were taken for study model, the sputum viscosity was detected by rotational viscometer, the expressions of TNF-α, AQP4 in bronchoalveolar lavage fluid were assessed by western blot analysis, and the histological changes in endothelium were evaluated by HE staining and scanning electron microscopy (SEM).

**Results:**

Study results revealed that tracheostomy gave rise to the increase of sputum viscosity, TNF-α and AQP4 expression, mucosa and cilia damage, yet the saline fluid nebulization could significantly decrease the changes of those indicators, besides, the hypertonic, isotonic and hypertonic saline nebulization produced different efficacy.

**Conclusions:**

Osmolality plays an important role in the saline fluid nebulization after tracheostomy, and 3% saline fluid nebulization seems to be more beneficial, further studies on the role of osmolality in saline fluid nebulization are warranted.

## Background

As a rather simple yet serious surgery, tracheostomy are widely conducted in intensive care units (ICUs) and emergency department, it is well documented that tracheostomy may shorten the duration of ventilation, reduce pulmonary morbidity, save critical care resource utilization and decrease the hospital length of stay [1–3], however, the trachea opening and tube insertion render the trachea directly exposing to the external environment, making the airway in dry condition and hard to clear the sputum, and finally result in ventilator-associated pneumonia [4–6], therefore, optimal managements of the airway are essential for patients with tracheostomy.

Nebulization of saline solution or drugs have been highly recommend by related guidelines and nursing experts [7–10], nevertheless, although numerous studies have focused on this area, their conclusions on the use of saline solution remain inconsistent. Several studies [11, 12] concluded that hypertonic saline might promote the sputum induction, reduce the incidence of complications, while Huang et al. [13, 14] found that hypotonic saline nebulization might be superior over isotonic saline in maintaining the airway patency and reducing pulmonary infection for patients with tracheostomy, interestingly, a recent study [15] found that hypertonic and isotonic saline nebulization offered no significant efficacy difference in patients with tracheostomy. So far, no studies have been performed to directly compare the efficacy of hypotonic, isotonic and hypertonic saline nebulization in clinic and lab settings.

The bronchoalveolar lavage fluid (BALF) are widely used for detecting biological molecules change in clinic and laboratory [16], and it have been reported that TNF-α and AQP4 in BALF are closely associated with the airway function, the elevated TNF-α expression in BALF may be related to inflammatory mechanism and causes ventilator-induced lung injury even high mortality [17], besides, increased inflammatory cytokines and AQP4 in BALF have been observed in the condition of hypobaric hypoxia [18], it’s noteworthy that even though TNF-α and AQP4 may related to the change of airway function, the role of TNF-α and AQP4 in the conditions of tracheostomy remain unclear, further studies on this issue are warranted.

Based on literatures review, we have found that even though the efficacy of saline solutions nebulization in patients with tracheostomy are proven, the effects of saline solutions with different osmolality remain conflicting, and to date no studies have directly compare the efficacy of hypotonic, isotonic and hypertonic saline fluid nebulization in patients with tracheostomy, further researches on the role of osmolality in saline nebulization are necessary.

Given the insufficiency of evidence on the role of osmolality in saline nebulization, and the optimal osmolality in saline nebulization to patients’ outcome is highly relevant, we performed this experimental study with the following objectives: (1) to discuss the biological changes in the early stage after tracheostomy; (2) to compare the efficacy of hypotonic, isotonic and hypertonic saline solutions nebulization on the airway after tracheostomy; and (3) to analyze the role of saline osmolality on the sputum viscosity, histological changes, expression of TNF-α and AQP4 in BALF.

## Methods

### Animals

The animal experimental protocols were approved by the Animal Care and Use Committee of Soochow University and complied with the Guide for the Care and Use of Laboratory Animals by the National Institutes of Health. Ninety seven male adult Sprague-Dawley rats (250–300 g) were purchased from Animal Center of Chinese Academy of Sciences (Shanghai, China) and housed in a light and temperature controlled environment with free access to food and water.

### Tracheostomy model and experimental design

**Fig. 1:**
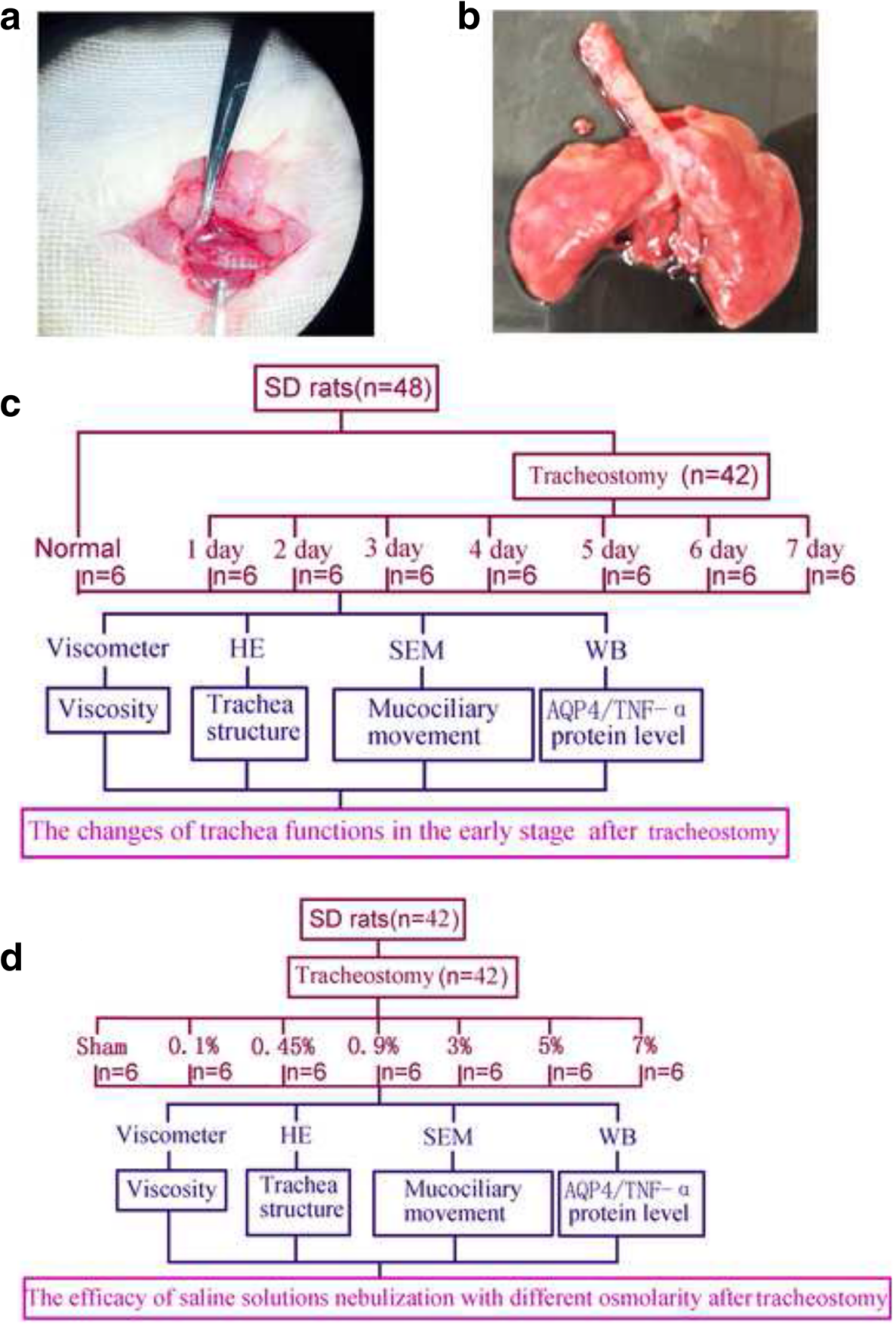
The methods and study design for this study (**a**): The exposure of trachea for tracheostomy under the microscopy. **b** Gross morphology of the trachea and lung specimen from SD rats. **c** Study design on trachea function changes in the early stage after tracheostomy (**d**): Study design on the efficacy of nebulization with saline solution of different osmolality after tracheostomy

All the tracheostomy procedures were attempted to be performed in the sterile environment and under the assistance of microscopy (ZEISS GX-3, German) (Fig. 1a), briefly, the animals were anesthetized with ketamine (20 mg/kg) intraperitoneally, the tracheal rings and tracheal cartilage were separated and exposed after cut off some portion of the skin tissue with a vertical neck incision, then a 0.8 cm incision was made at the 3–4 airway cartilage, further a stoma in size of 1/2 ~ 2/3 airway wall was made, finally the surrounded skin tissues were sutured into the inner wall of trachea to form a permanent tracheal stoma. After related interventions were done, the rats were euthanized with ketamine (60 mg/kg) intraperitoneally, then the airway and lung were excised for further analysis (Fig. 1b).

The experiments were mainly divided into two parts. In part one, 48 rats (six rats for each group) were randomly distributed to normal group and seven experimental groups arranged by time: 24, 48 and 72 h, 4, 5, 6, 7 days after tracheostomy (Fig. 1c). In part two, we implemented sham-operated rats (sham group, *n* = 6) as controlled group, while the intervention groups were randomly separated into 6 groups, which underwent 0.1% (0.1% group, *n* = 6), 0.45% (0.45% group, *n* = 6), 0.9% (0.9% group, *n* = 6), 3% (3% group, *n* = 6), 5% (5% group, *n* = 6) and 7% (7% group, *n* = 6) saline solutions nebulization after tracheostomy (Fig. 1d). All intervention groups received corresponding nebulization interventions once per day for a week. During our experimental period, no rats had died in the part one, yet two rats died in the part two, a rat in 0.45% group died in the fourth day after tracheostomy, a rat in 0.9% group died in the sixth day after tracheostomy, and we had made necessary supplements to ensue enough sample.

### Nebulization

For every nebulization operation, rats were anesthetized with intraperitonal euthanized with 4% chloral hydrate, then a 0.2 cm-diameter soft tube was connected to a *flexiVent* (Scarsdale, New York, USA) gas-driven jet nebulizer, the other end were well placed at the opening of stoma, a tidal volume of 10 ml/kg at 180 breaths/min with 3-cmH_2_O positive end-expiratory pressure was set according to the producer instruction, a total of 10 ml nebulized saline solution for each concentration was made and administrated instantly.

### Viscosity measurement

Rotational viscometer (NXE 1, Cheng Dou, China) was adopted for detecting the sputum viscosity, with consideration to the special characteristics of airway flow, we chose the IOOS model as test condition in the guide of company instruction. For part one, sputum specimens were examined daily for ascertaining the viscosity changes after tracheostomy, and for part two, the sputum specimens were measured after a weekly-long nebulization intervention. Every measurement has been repeated three times for data collection, and the average value has been calculated for further data analysis.

### Hematoxylin and eosin (HE) staining

A 0.5 cm long sample of tracheal tissue was excised nearly from the 0.3 to 0.8 com trachea portion lower the stoma, and was fixed in 2.5% glutaraldehyde for 24 h, the specimens were dehydrated and then embedded in paraffin, finally the transverse section of specimens were sliced for HE staining as previously described [19, 20], each tissue section was examined and photopgraphed using an Olympus light microscope (Olympus OX51), Image J software (US National Institutes of Health, Bethesda, MD, USA) was used to calculate the white blood cell counts, we tended to quantify the disarrangement of mucosal epithelium or basement membrane yet no accurate method to resort.

### Scanning electron microscopy (SEM)

Another 0.5 cm long sample of tracheal tissue linking to the specimen for HE staining was obtained for SEM measurement. The specimens were fixed in 2.5% glutaraldehyde (Santacruze, Shanghai, China) for 24 h at 4 °C and further processed for 24 h in 0.1 M sodium cacodylate solution. Afterwards, the specimens were dried with increasing concentrations of acetone for 70 min. After critical-point drying using 100% acetone and carbon dioxide, the specimens were fixed on plates and coated with a 20 nm layer of gold in a sputter-coater (E 5400 Polaron, Quorum Technologies, Newhaven, UK). Topographical examination was performed with a scanning electron microscope (HITACHI TM3030, Japan) at an accelerating voltage of 15 kV. The surface morphology of all SEM specimens was evaluated using ImageJ to determine the total lumen-facing area of each specimen and its endothelial area.

### Western blot

Bronchoalveolar lavage fluid (BALF) was obtained by instilling 1 ml PBS through the tracheal cannula and suctioning back with a volume of 0.8–0.9 ml. The BALF was centrifuged at 12,000 g for 20 min at 4 °C as soon as finish the collection, and the supernatant was stored at −80 °C. The protein concentration was measured by the bicinchoninic acid (BCA) method using enhanced BCA protein assay kit (Beyotime, Shang Hai, China). The samples were separated using 10% SDS-PAGE and electro-transferred onto nitrocellulose membrane (Bio-Rad, USA). The membranes were blocked with 5% non-fat milk for 1 h at room temperature, and were then incubated with primary antibodies in 5% BSA (in TBS + 0.1%Tween 20) overnight at 4 °C. The β-tublin (1:3000,Santa cruz, USA) was used as a loading control. The membranes were washed three times for 5 min each in TBS + 0.1%Tween 20, and were then incubated in the appropriate horseradish peroxidase (HRP)-conjugated secondary antibodies (Santa cruz, USA) for 2 h at room temperature. Finally, the protein bands were visualized using enhanced chemiluminescence (ECL). The relative quantity of proteins was analyzed using Image J and normalized to that of loading controls. Antibodies included: rabbit anti-TNF-α (1:1000, Abcam, USA), mouse anti-AQP4 (1:1000, Abcam, USA), rabbit anti -β-tublin (1:1000, Santa cruz, USA).

### Statistical analysis

All data were presented as the means ± SD. GraphPad Prism 5 (GraphPad Software Inc, USA) was adopted for all statistical analysis. Data were analyzed using one-way or two-way ANOVA analysis, a value of *P* < 0.05 was considered statistically significant. All evaluations were performed by investigators blinded to the experimental groups.

## Results

### Sputum viscosity and TNF-α, AQP4 expressions increased gradually after tracheostomy

**Fig. 2:**
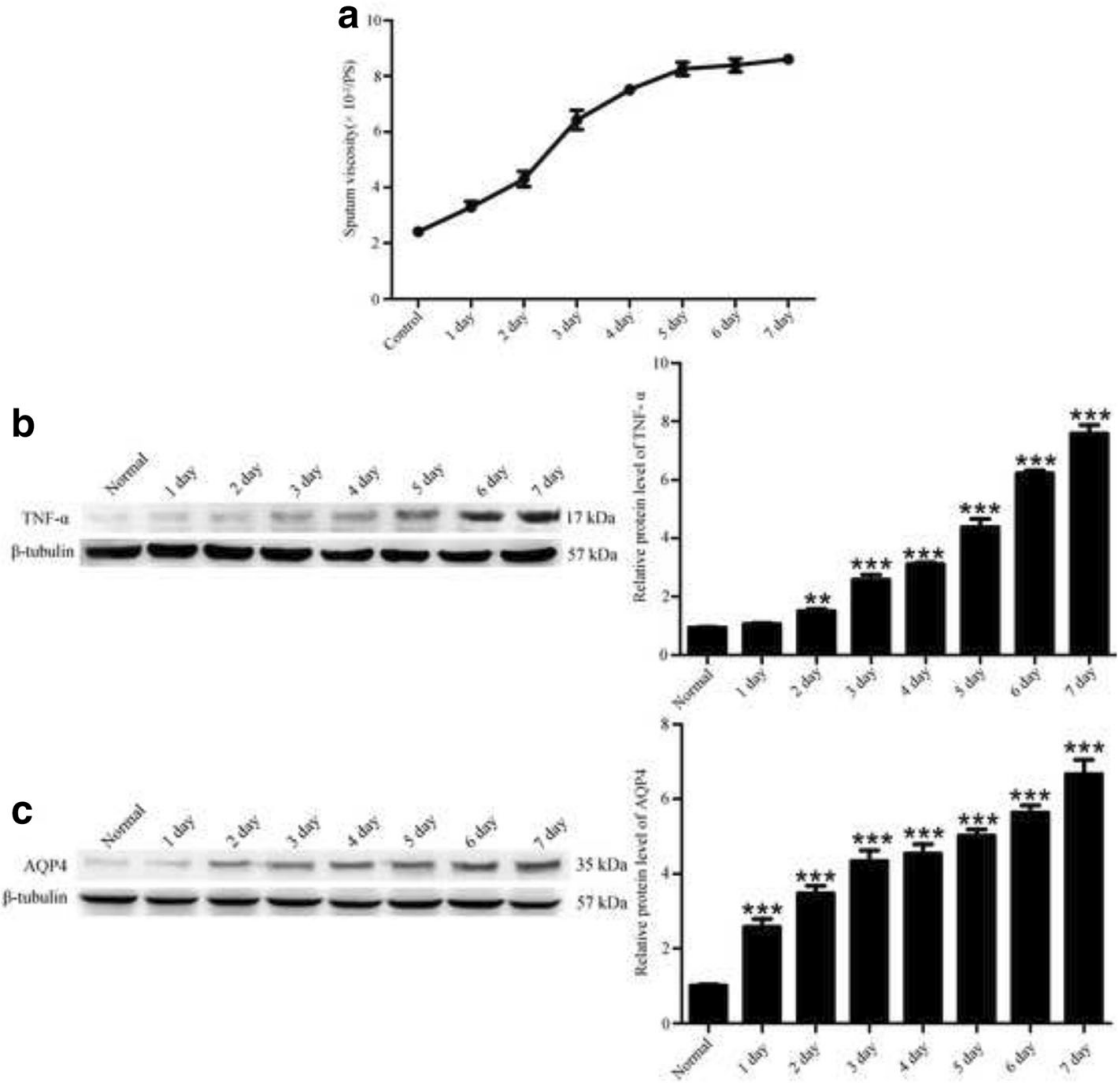
Sputum viscosity and TNF-α, AQP4 expressions in the early stage after tracheostomy (**a**): tracheostomy— induced sputum viscosity changes in the early stage after tracheostomy. **b** Western blot analysis on the TNF-α, AQP4 expression changes within a week after tracheostomy (all values are means ± SEM, **p < 0.01 vs. normal and **p < 0.001 vs. Normal)

Significant changes of sputum viscosity were observed in different time point compared to the normal group after tracheostomy (Fig. 2a), and in day five reached a plateau stage. TNF-α, AQP4 expressions gradually increased and peaked at 24 h peaked at day seven after tracheostomy (Fig. 2b and c).

### Trachea mucosa and cilia damage increased in the early stage after tracheostomy

**Fig. 3:**
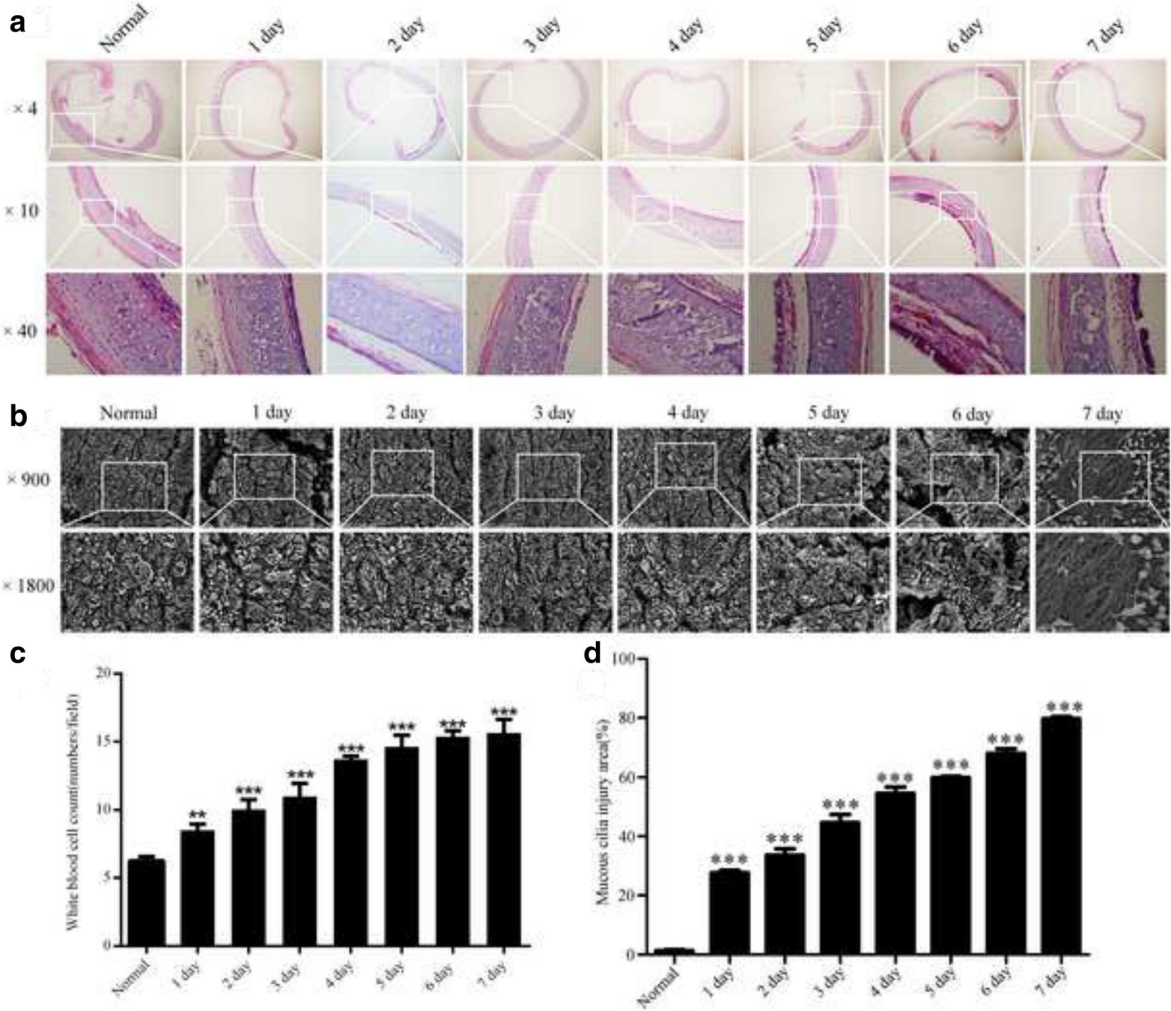
HE staining and SEM assay on the histological changes in the early stage after tracheostomy (**a**): HE staining analysis on trachea structure changes within a week after tracheostomy. **b** SEM analysis on the changes of cilia and mucosa in the early stage after tracheostomy. **c** The changes on the white blood cell counts in HE staining (**p < 0.01 and ***p < 0.001 vs. Normal). **d** The changes on the damage area of cilia and mucosa in the SEM a (***p < 0.001 vs. Normal)

HE staining assay showed that the trachea structure changed significantly after tracheostomy, with the time went by, the necrosis of basic epithelial cells and exposure of basement membrane were more severe, and neutrophil infiltration of mucosa, even hemorrhage were observed (Fig. 3a), with time went by, the white blood cell count increased gradually (Fig. 3c). Similarly, significant cilia and mucosa damage in SEM were observed in the early stage after tracheostomy (Fig. 3b), the cilia and mucosa damage became severe if no invention has made (Fig. 3d).

### Saline nebulization after tracheostomy decreased sputum viscosity and reduced TNF-α, AQP4 expressions

**Fig. 4:**
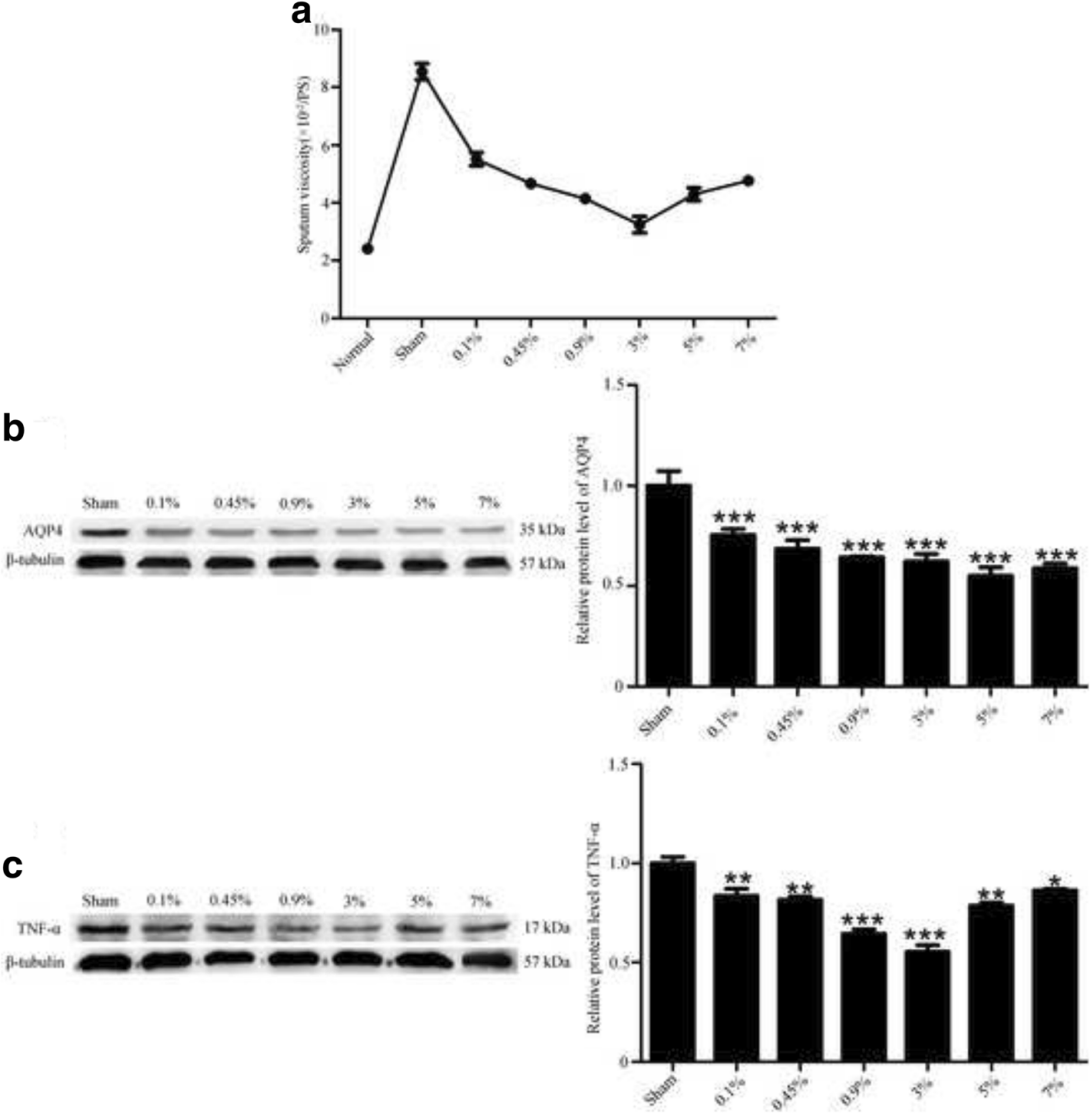
Sputum viscosity and TNF-α, AQP4 expressions after nebulization intervention (**a**): Sputum viscosity changes after nebulization intervention. **b** Western blot analysis on the TNF-α expression changes after nebulization intervention (all values are means ± SEM *p < 0.05 **p < 0.01 vs. and ***p < 0.01 vs. sham). **c**: Western blot analysis on the AQP4 expression changes after nebulization intervention (all values are means ± SEM, *p < 0.05 **p < 0.01 and ***p < 0.001 vs. Sham)

Viscosity test results revealed that nebulization could significantly decreased the sputum viscosity after tracheostomy, yet saline nebulization with different osmolality might exert different efficacy, among then the 3% saline solution seemed to be most potent in reducing the sputum viscosity (Fig. 4a). Interestingly, the protein level of TNF-α, AQP4 in BALF were correspondingly decreased after nebulization intervention, as the trend of viscosity, the TNF-α expressed lowest in 3% saline nebulization group (Fig. 4b), but the AQP4 expression did not show any significant change among intervention groups (Fig. 4c).

### Saline nebulization after tracheostomy attenuated the trachea mucosa and cilia damage

**Fig. 5:**
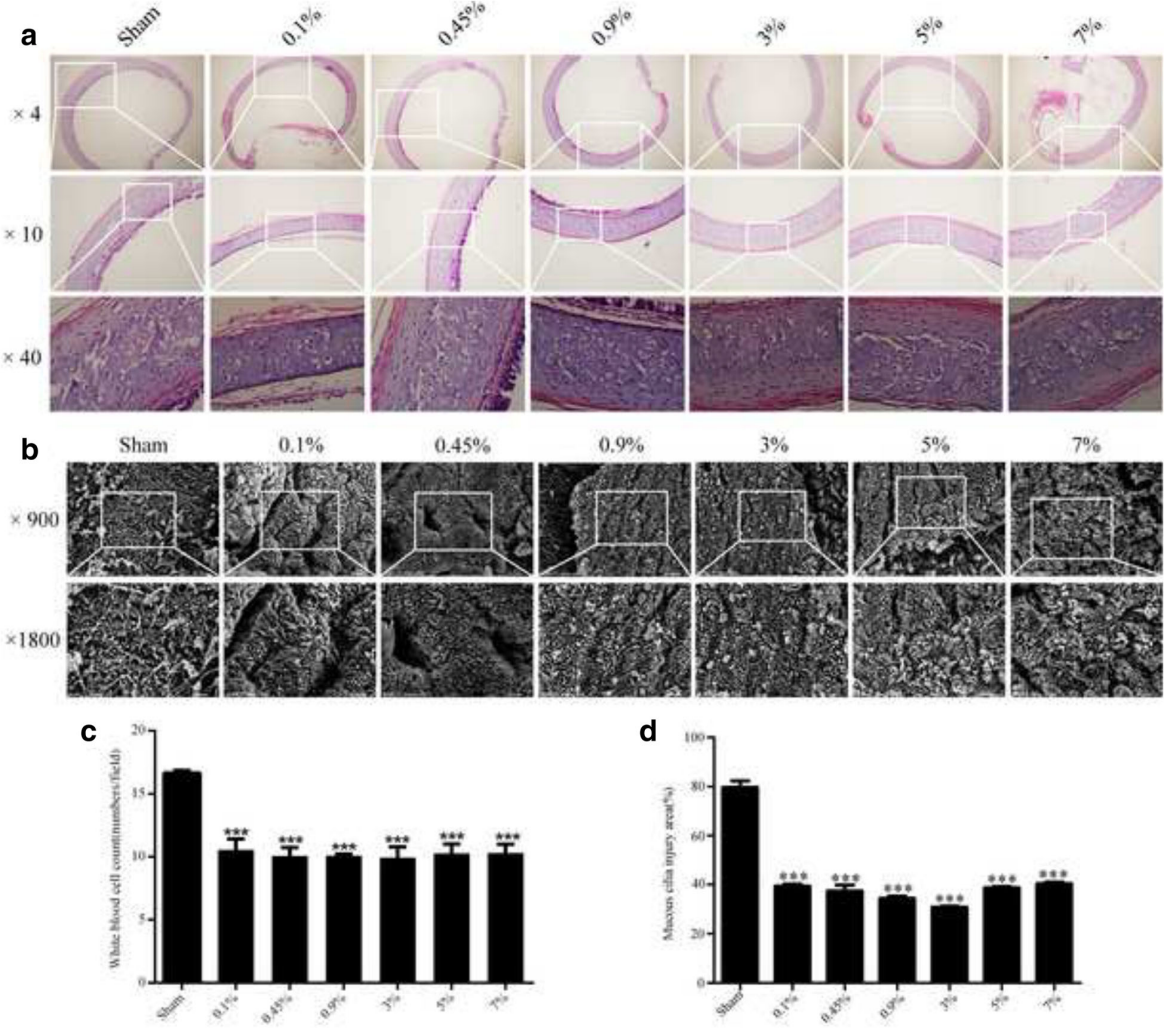
HE staining and SEM assay on the histological changes after nebulization intervention (**a**) HE staining analysis on trachea structure changes within a week after tracheostomy. **b** SEM analysis on the changes of cilia and mucosa in the early stage after tracheostomy (*p < 0.001 vs. Sham). **c** The changes on the white blood cell counts in HE staining after intervention (***p < 0.01 vs. Sham). **d** The changes on the damage area of cilia and mucosa in the SEM after intervention (***p < 0.001 vs. Sham)

HE staining assay indicated that saline nebulization might improve the mucosa function, reduce the disorder of trachea structure, and keep epithelium mainly intact (Fig. 5a), particularly, saline nebulization produced significant reduction of white blood cell counts, yet no significant difference was observed in the white blood cell counts between nebulization groups (Fig. 5c). Consistently, SEM results showed that nebulization could attenuate the cilia damage caused by tracheostomy (Fig. 5b), saline nebulization interventions seemed to improve the adhesive and lodging situation of cilia, reduce the exposure of trachea base membrane, and decrease the loss of cilia. However, no significant differences were found among different intervention groups (Fig. 5d).

## Discussion

In this study, we come to the finding that tracheostomy lead to the increase of sputum viscosity, activation of TNF-α and AQP4, disorder of trachea mucosa and cilia, as expected, saline nebulization might provide a beneficial effect on reducing the sputum viscosity, decreasing the TNF-α and AQP4 expression, promoting the function of trachea mucosa and cilia, our results are consistent with previous studies [21–24]. Besides, the efficacy of saline nebulization with different osmolality after tracheostomy might be different.

Previous study have proven that the long-term ultrasonic nebulization of saline may increase the influx of inflammatory cells and oxidative damage, and promote the change of lung architecture in normal rats [25], yet no similar studies on the effects of saline nebulization on rats undergone tracheostomy have been published. Different from Campos’s study, our studies focused on the short-term change of airway function in rats undergone tracheostomy, also, in our study we have designed the normal and sham group as control group to discuss the efficacy of nebulization. To the best of our knowledge, this paper is the first one to investigate the role of saline fluid with different osmolality on rats, in hoping that may provide some evidence for clinic nebulization.

Generally, the biological production and removal of sputum are crucial to the normal airway function, nevertheless, as an invasive operation, tracheotomy may not only result in certain mechanical injury, but also expose the trachea to the outer air environment with compromise of heating and humidification function of upper respiratory tract, the dry air condition and more outer stimulus into the trachea may cause dry and vulnerable circumstances requiring more mucus secretion, therefore the sputum viscosity increase and the sputum may hard to be expectorated. Our study find that sputum viscosity increase gradually in the early stage after tracheostomy, and nebulization exert positive effects on reducing the sputum viscosity, and the 3% saline nebulization seem to be most potent. Bilton et al. [26] has noted that the change of sputum viscosity depends on the degree of mucin glycosylation, the hypertonic saline solution may reduce the sputum viscosity by decreasing the total amount of sugar chains woven into a network, but this effect may be saturated at 3% saline solution. Nevertheless, the specific mechanism on osmolality of saline nebulization reducing the sputum viscosity remain unclear and warrants further study.

It’s well-known that pathogenic bacteria may easily invade into the trachea after tracheostomy, lead to a higher level of inflammation. The early modification of interleukin-1 and TNF-α in the drainages may best predict surgical-site infection [21]. The results from Goldman’ study [27] suggested that TNF-α might mediate the acid aspiration-induced local and systemic injury, specifically the systemic leukosequestration and permeability following localized aspiration was mediated by TNF-α induced synthesis of an adhesion protein, and the tracheostomy might promote the up-regulation of neutrophil endothelial adhesion receptors. After tracheostomy, the increase of TNF-α level may cause endothelial cell damage and hence increase permeability, and induce neutrophil transmigration through the vascular endothelial membrane [28]. The nebulization may improve the endothelial membrane function and reduce the TNF-α expression. Interestingly, the TNF-α level reaches the lowest at the 3% saline nebulization group, therefore, the 3% saline solution may optimally improve the endothelial membrane function, the saline solution with higher osmolality may have too strong effects on endothelial membrane to be helpful.

The water-transporting properties of the airways have been believed to be essential for the humidification of inspired air and maintaining the volume and composition of the airway surface liquid (ASL). Measurements of osmotic water permeability by previous studies [29, 30] indicate that large and small airways have moderately high osmotic water permeability, and AQP4 are widely involved in the upper and lower airway epithelia function. Comparison of humidification efficiencies from wild-type and double knockout mice lacking AQP4 showed a small but significant impairment in lower airway humidification [23]. In our study, AQP4 increased gradually after tracheostomy, and nebulization may significantly reduce the AQP4 expressions in BALF, the improvement of water permeability after nebulization may explain this. Similarly, the hypertonic saline solution have more advantages in ameliorating the water permeability after tracheostomy, the depth and ionic composition of the ASL depend on the ion transporting properties of the airway epithelium and the rate of evaporative water loss, the hypertonic saline may produce stronger influence on the ion transporting properties [31].

There is no doubt that tracheostomy may cause injury to mucosa and cilia, and our result have suggested the protective effects of nebulization on trachea mucosa and cilia, there is approximately 10% difference in the cilia injury area between intervention groups, but this did not reach statistical significance, and again 3% saline nebulization seems to be more advantageous in reducing cilia injury area and maintaining integrity of mucosa. The osmolality of saline solution may play a key role in this difference, a human study conducted by Wabnitz et al. [32] has shown that hypertonic saline stimulates ciliary beat frequency (CBF) compared with isotonic saline in vitro, extracellular sodium is believed to specifically and competitively inhibit an adenosine triphosphate-gated channel involving calcium influx, which in turn can prompt ciliary motility, while excessive sodium within airway surface fluid may inhibit this channel [33], this may explain the results that higher osmolality like 5% and 7% produce less beneficial outcome.

In recent years, considerable interest has been focused on the delivery techniques or category of nebulization, yet very few studies focus on the osmolality of solution for nebulization, our study provide significant sights into this issue, and we find that 3% saline solution maybe a better option for nebulization with respect to the advantages in related indicators. Those who uphold the hypotonic saline solution argued that the evaporation of fluid during nebulization might concentrate the hypotonic fluid, making the hypotonic particles into isotonic one, which is more in conformity to the biological condition [13, 14], yet no definite evidence supports this. Contrarily, studies [34, 35] from laboratory revealed that either isotonic or hypertonic saline solution produced a negative impact on CBF, and concluded that hypertonic saline might also disrupt nasal epithelial cells. The results on the role of saline osmolality in nebulization still remain inconclusive, and this issue warrants further study.

Several limitations in this study should be addressed. Firstly, even though multiple methods have been used for detecting the biological changes after tracheostomy, no direct measurement on the CBF has been made. Secondly, the groups designed for nebulization intervention are rather limited, it’s difficult to identify the optimal saline osmolality for nebulization, therefore, more specific groups design are needed in future study. Thirdly, we only have focused on the biological changes in the early stage after tracheostomy, long-term effects of nebulization after tracheostomy need further investigation.

## Conclusions

In conclusion, we find that tracheostomy can cause significant damage to the biological functions of trachea, and saline nebulization after tracheostomy is quite necessary and beneficial, although it’s hard to make any definite conclusions on the effects of saline nebulization with different osmolality after tracheostomy, based on our results, 3% saline solution seems to be a better option with consideration to its advantages in reducing sputum viscosity, combating inflammation and maintaining mucosa integrity and decreasing cilia injury. However, we would suggest that animal study provide limited applicability to the clinical situation, large-scale RCTs on the role of osmolality for nebulization after tracheostomy should be conducted.

## Abbreviations

ASL: Airway surface liquid
BALF: Bronchoalveolar lavage fluid
CBF: Ciliary beat frequency
ICUs: Intensive care units
SEM: Scanning electron microscopy

## References

[1] Mehta AB, Cooke CR, Wiener RS, Walkey AJ. Hospital Variation in Early Tracheostomy in the United States: A Population-Based Study. Crit Care Med. 2016;44(8):1506–14. 10.1097/CCM.0000000000001674 PMC4949074 27031382

[2] Hyde GA, Savage SA, Zarzaur BL, Hart-Hyde JE, Schaefer CB, Croce MA, Fabian TC (2015). Early tracheostomy in trauma patients saves time and money. Injury.

[3] Sofi K, Wani T (2010). Effect of tracheostomy on pulmonary mechanics: An observational study. Saudi J Anaesth.

[4] Campisi P, Forte V (2016). Pediatric tracheostomy. Semin Pediatr Surg.

[5] Marshall RV, Haas PJ, Schweinfurth JM, Replogle WH. Tracheotomy Outcomes in Super Obese Patients. JAMA Otolaryngol Head Neck Surg. 2016;142(8):772–6. 10.1001/jamaoto.2016.1089 27228561

[6] Edwards JD, Houtrow AJ, Lucas AR, Miller RL, Keens TG, Panitch HB, Dudley RA. Children and Young Adults Who Received Tracheostomies or Were Initiated on Long-Term Ventilation in PICUs. Pediatr Crit Care Med. 2016;17(8):e324–34. 10.1097/PCC.0000000000000844 PMC5113027 27367044

[7] Colandrea M, Eckardt P (2016). Improving Tracheostomy Care Delivery: Instituting Clinical Care Pathways and Nursing Education to Improve Patient Outcomes. ORL Head Neck Nurs.

[8] Dawson D (2014). Essential principles: tracheostomy care in the adult patient. Nurs Crit Care.

[9] Paul F (2010). Tracheostomy care and management in general wards and community settings: literature review. Nurs Crit Care.

[10] Dres M, Ferre A, Becquemin MH, Dessanges JF, Reychler G, Durand M, Escabasse V, Sauvaget E, Dubus JC (2012). [Inhalation therapy: provocation tests, infectious risks, acute bronchiolitis and ENT diseases. GAT aerosolstorming, Paris. Rev Mal Respir.

[11] Gupta HV, Gupta VV, Kaur G, Baidwan AS, George PP, Shah JC, Shinde K, Malik R, Chitkara N, Bajaj KV (2016). Effectiveness of 3% hypertonic saline nebulization in acute bronchiolitis among Indian children: A quasi-experimental study. Persp Clin Res.

[12] Gupta S, Ahmed F, Lodha R, Gupta YK, Kabra SK (2012). Comparison of effects of 3 and 7% hypertonic saline nebulization on lung function in children with cystic fibrosis: a double-blind randomized, controlled trial. J Trop Pediatr.

[13] Huang H, Li C, Wu Y (2007). Application of oxygen jet nebulization with 0.45% saline in tracheotomized patients. J Nurs Sci.

[14] Li L, Zhou F, Sang X, Huang Y (2014). Comparative study of effect of different airway humidification fluid in tracheotomy patients. Med Innovation China.

[15] Li S, Lu Q, Deng H, Pang S (2016). Effect of different saline solutions on sputum inducing in patients with tracheostomy. J Nurs Training.

[16] Eigl S, Hoenigl M, Spiess B, Heldt S, Prattes J, Neumeister P, Wolfler A, Rabensteiner J, Prueller F, Krause R, et al. Galactomannan testing and Aspergillus PCR in same-day bronchoalveolar lavage and blood samples for diagnosis of invasive aspergillosis. Med Mycol. 2016;23(10):641–9. 10.1093/mmy/myw102 27744310

[17] Yang SH, Lin JC, Wu SY, Huang KL, Jung F, Ma MC, Wang Hsu GS, Jow GM (2015). Membrane translocation of IL-33 receptor in ventilator induced lung injury. PLoS One.

[18] Wang C, Yan M, Jiang H, Wang Q, Guan X, Chen J, Wang C (2016). Protective effects of puerarin on acute lung and cerebrum injury induced by hypobaric hypoxia via the regulation of aquaporin (AQP) via NF-kappaB signaling pathway. Int Immunopharmacol.

[19] Zhang WG, He L, Shi XM, Wu SS, Zhang B, Mei L, Xu YJ, Zhang ZX, Zhao JP, Zhang HL (2014). Regulation of transplanted mesenchymal stem cells by the lung progenitor niche in rats with chronic obstructive pulmonary disease. Respir Res.

[20] Nakajima H, Nakajima HO, Dembowsky K, Pasumarthi KB, Field LJ (2006). Cardiomyocyte cell cycle activation ameliorates fibrosis in the atrium. Circ Res.

[21] Candau-Alvarez A, Gil-Campos M, De la Torre-Aguilar MJ, Llorente-Cantarero F, Lopez-Miranda J, Perez-Navero JL (2015). Early modification in drainage of interleukin-1beta and tumor necrosis factor-alpha best predicts surgical-site infection after cervical neck dissection for oral cancer. J Oral Maxillofac Surg.

[22] Okuyucu S, Akoglu E, Babayigit C, Akoglu S, Dagli S (2009). Ex vivo tracheal mucociliary clearance in rats: comparisons of nebulization versus irrigation with lactated ringer and saline solutions. J Otolaryngol Head Neck Surg.

[23] Song Y, Jayaraman S, Yang B, Matthay MA, Verkman AS (2001). Role of aquaporin water channels in airway fluid transport, humidification, and surface liquid hydration. J Gen Physiol.

[24] Ruddy MK, Drazen JM, Pitkanen OM, Rafii B, O’Brodovich HM, Harris HW (1998). Modulation of aquaporin 4 and the amiloride-inhibitable sodium channel in perinatal rat lung epithelial cells. Am J Physiol.

[25] Campos KK, Leal SF, Costa DC, de Lima WG, Bezerra FS (2015). Long-term exposure to ultrasonically nebulized distilled water and saline causes cellular influx and oxidative stress in lung tissue of rats. Exp Lung Res.

[26] Bilton D, Stanford G (2014). The expanding armamentarium of drugs to aid sputum clearance: how should they be used to optimize care?. Curr Opin Pulm Med.

[27] Goldman G, Welbourn R, Kobzik L, Valeri CR, Shepro D, Hechtman HB (1990). Tumor necrosis factor-alpha mediates acid aspiration-induced systemic organ injury. Ann Surg.

[28] Lewis CA, Martin GS (2004). Understanding and managing fluid balance in patients with acute lung injury. Curr Opin Crit Care.

[29] Matsui H, Davis CW, Tarran R, Boucher RC (2000). Osmotic water permeabilities of cultured, well-differentiated normal and cystic fibrosis airway epithelia. J Clin Invest.

[30] Farinas J, Kneen M, Moore M, Verkman AS (1997). Plasma membrane water permeability of cultured cells and epithelia measured by light microscopy with spatial filtering. J Gen Physiol.

[31] Verkman AS, Matthay MA, Song Y (2000). Aquaporin water channels and lung physiology. Am J Physiol Lung Cell Mol Physiol.

[32] Wabnitz DA, Wormald PJ (2005). A blinded, randomized, controlled study on the effect of buffered 0.9% and 3% sodium chloride intranasal sprays on ciliary beat frequency. Laryngoscope.

[33] Ma W, Korngreen A, Uzlaner N, Priel Z, Silberberg SD (1999). Extracellular sodium regulates airway ciliary motility by inhibiting a P2X receptor. Nature.

[34] Boek WM, Keles N, Graamans K, Huizing EH (1999). Physiologic and hypertonic saline solutions impair ciliary activity in vitro. Laryngoscope.

[35] Min YG, Lee KS, Yun JB, Rhee CS, Rhyoo C, Koh YY, Yi WJ, Park KS (2001). Hypertonic saline decreases ciliary movement in human nasal epithelium in vitro. J Otolaryngol Head Neck Surg.

